# “Epigenetic Memory” Phenomenon in Induced Pluripotent Stem Cells

**Published:** 2013

**Authors:** E.A. Vaskova, A.E. Stekleneva, S.P. Medvedev, S.M. Zakian

**Affiliations:** 1Institute of Cytology and Genetics, Siberian Branch, Russian Academy of Sciences, prosp. Akad. Lavrentyeva, 10, Novosibirsk, Russia, 630090; Meshalkin State Research Institute of Circulation Pathology, Rechkunovskaya Str., 15, Novosibirsk, Russia, 630055; Institute of Chemical Biology and Fundamental Medicine, Siberian Branch, Russian Academy of Sciences, prosp. Akad. Lavrentyeva, 8, Novosibirsk, Russia, 630090

**Keywords:** pluripotency, reprogramming, epigenetics

## Abstract

To date biomedicine and pharmacology have required generating new and more consummate models. One of the most perspective trends in this field is using induced pluripotent stem cells (iPSCs). iPSC application requires careful high-throughput analysis at the molecular, epigenetic, and functional levels. The methods used have revealed that the expression pattern of genes and microRNA, DNA methylation, as well as the set and pattern of covalent histone modifications in iPSCs, are very similar to those in embryonic stem cells. Nevertheless, iPSCs have been shown to possess some specific features that can be acquired during the reprogramming process or are remnants of epigenomes and transcriptomes of the donor tissue. These residual signatures of epigenomes and transcriptomes of the somatic tissue of origin were termed “epigenetic memory.” In this review, we discuss the “epigenetic memory” phenomenon in the context of the reprogramming process, its influence on iPSC properties, and the possibilities of its application in cell technologies.

## INTRODUCTION

Organism cells of any type have individual epigenomes: certain set and pattern of posttranslational covalent histone modifications and DNA methylation, and the presence of specific small non-coding RN As. The combination of these factors forms a unique chromatin structure, which is inherent to cells of a special type.

Chromatin of pluripotent cells usually stays in the decompacted state and open configuration [1, 2]. Such a configuration promotes a dynamic posttranslational remodeling of histones and DNA methylation/demethylation processes during cell differentiation and specialization [3, 4]. The pluripotent cells also contain bivalent domains (i.e., the areas enriched in markers of both active and inactive chromatin together). Most bivalent domains are associated with the transcription start sites of the genes involved in the development. For example, bivalent domains were found in the genes of early mouse development (*Sox1, Pax3, Msx1, *and* Irx3*). A low transcriptional level is typical of these genes in pluripotent cells, while during differentiation the bivalent domains are converted into monovalent ones with markers of either active or inactive chromatin; therefore, genes are either activated or suppressed, providing a certain type of cell specialization [1].

Two types of pluripotent cells are widely used in biomedicine today: embryonic stem cells (ESCs) and induced pluripotent stem cells (iPSCs). iPSCs are derived from somatic cells via ectopic overexpression of certain transcription factors, including *Oct4, Sox2, Klf4, c-Myc, Nanog, *and *Lin28*, or microRN As [5-8]. iPSCs can be obtained at any period of human life and from various somatic cells (skin fibroblasts, keratinocytes, adipose stem cells, cells of peripheral blood, etc.); they are donor-specific (autologous). These are the reasons why the use of iPSCs is the preferred strategy in biomedicine and why a detailed, large-scale study of their properties and scope of clinical use is an urgent problem.

Based on today’s knowledge, iPSCs and ESCs are known to have virtually the same properties: they express similar sets of genes and form teratomas containing the derivatives of all three germ layers. Mouse iPSCs at tetraploid complementation are capable of forming chimeras and generating valid organisms [9]. Meanwhile, plenty of studies have produced evidence that the lines of iPSCs acquire a variety of genetic and epigenetic aberrations, including impaired functioning of imprinted genes, changed numbers of gene copies, point mutations, aberrant patterns of DNA methylation, etc. during the reprogramming process [10-14]. At that, both the aberrations acquired during reprogramming and some retained epigenetic markers of somatic cells cause differences in the epigenomes and transcriptomes of ESCs and iPSCs. This phenomenon of inheritance of the initial somatic epigenomes and transcriptomes by iPSCs is known as epigenetic memory [15-17].

An analysis of the identity of epigenomes and transcriptomes among iPSCs and their progenitor cells, the effects of epigenetic memory on iPSC properties, and the possibilities for its practical application in biomedicine are the main issues touched upon in this review.

## THE EPIGENETIC MEMORY PHENOMENON IN THE PROCESS OF SOMATIC CELLS REPROGRAMMING TO THE PLURIPOTENT STATE

Advanced methods of high-performance analysis have proved the similarity of gene expression profiles, set, and distribution patterns of histone covalent modifications, DNA methylation, and microRN A expression in iPSCs and ESCs. However, minimal differences exist in their transcriptomes and epigenomes. Different patterns of DNA methylation in independent iPSC lines have been analyzed in a number of recent studies, cytosine methylation of DNA CpG-nucleotides being the most explored phenomenon [18]. CpG nucleotides can be scattered through a genome or concentrated in special regions known as CpG islands. The CpG islands typically reside near the gene promoters, and the high level of promoter methylation correlates with gene repression [19]. K. Kim *et al. *[16] analyzed, using Comprehensive High-throughput Array-based Relative Methylation (CHARM) analysis, DNA methylation patterns both in ESCs and iPSCs derived from two different somatic cell types: mouse hematopoetic progenitors and tail-tip fibroblasts. This approach allowed the authors to assess the methylation of approximately 4.6 million CpG nucleotides, including virtually all CpG islands and the adjacent areas but ignoring non-CpG methylation. Relative to ESCs, 3,349 differentially methylated regions (DMRs) were found in fibroblast-derived iPSCs, while only 516 were found in blood-derived ones. Notably, the CHARM analysis of the 24 mostly expressed DMRs has shown these regions to be associated with the genes involved in hemopoiesis (11 genes) and osteogenesis (3 genes). Thus, these results indicate that the genes initially responsible for cell specialization remain underreprogrammed during the reprogramming of an iPSC. The markers of skeletal musculature cells, *Cxcr4 *and* Integrin B1*, are significantly expressed in iPSCs derived from mouse skeletal muscle precursors, while granulocyte markers, *Lysozyme *and *Gr-1*, are expressed in iPSCs from granulocytes. 1,388 differentially expressed genes were found by comparing the transcriptional profiles of two iPSC lines. At that, the results of the bioinformation analysis of 100 genes with the maximum different expression levels allowed the authors to distribute them into groups of the genes involved in myofibrils and contactile fibers, muscule development, and β-cell and leucocyte activation [17]. Thus, these findings again attest to the epigenetic memory of iPSCs, in the form of retention of some specific traits of the initial somatic epigenomes and transcriptomes.

A similar phenomenon is also known in human iPSCs. K. Nishino *et al*. [20] performed a comparative analysis of DNA methylation in 5 lines of human ESCs, 22 iPSC lines, and 6 lines of initial somatic cells. Embryonic lung fibroblasts, amniotic and endometric cells, cells of umbilical vein epithelium and menstrual blood, and skin fibroblasts were used as somatic progenitor cells. Methylation was analyzed using DNA Illumina’s Infinium HumanMethylation27 BeadChip, with probes to 24,273 CpG sites within 13,728 genes. The methylation patterns of ~90% of CpG sites (17,572 sites) were similar in ES, iPS, and initial somatic cells, attesting to the fact that only 10% of CpG sites undergo modification and ensure the epigenetic variability of different types of cells. The comparison of pluripotent (ESC, iPSC) and initial somatic cells revealed 220 DMRs, 174 (79.5%) of which were hypermethylated in ESCs and iPSCs. These regions were associated mainly with the genes of transcription regulation. Interestingly, most of the hypomethylated DMRs localized within the CpG islands, while most of the hypermethylated DMRs resided beyond them. A comparison of DNA methylation in ESCs and iPSCs demonstrated that DMR numbers vary among the lines. In total, when a DMR was found in at least one of the iPSC lines under examination, 1,459 DMRs were found within 1,260 genes. Of special note, the DMR number is a totality of first the aberrant de novo methylated sites and, second, the sites inherited from the somatic cells of the initial types [20].

In addition, DNA methylation in human ESCs and iPSCs from neonatal umbilical blood (from two independent donors) was examined [21]. Consistent with the other studies, variation of the DNA methylation patterns among different lines was demonstrated, using a DNA-microchip including 5.2 million CpG sites that involved virtually all CpG islands and near sequences. At that, 267 of the 370 DMRs were acquired de novo as a result of reprogramming, while 75 were inherited by the epigenetic memory [21].

The studies described in [20] and [21] were performed using DNA microchips that allowed one to assess the genome-scale DNA methylation. However, advanced methods of molecular and genetic analysis allow a much more accurate and high-resolution examination of a cell’s epigenome. For example, R. Lister *et al*. [22] used the highly sensitive MethylC-Seq method to compare the methylomes of several iPSC lines derived from somatic cells of various types using various approaches. The method allows to assess cytosine methylation at the entire genome level with nucleotide resolution. The examined iPSCs included iPSCs derived from adipocytes using transduction by retroviruses carrying cDNAs of the *OCT4, SOX2, KLF4 *and *MYC *genes; iPSCs obtained using transduction by lentiviruses carrying cDNAs of the *OCT4, SOX2, NANOG *and *LIN28 *genes; IMr90 lung fibroblasts, and three iPSC lines obtained from foreskin fibroblasts using unintegrated episome vectors. The methylation status of 75.7-94.5% of cytosine residues was assessed in all lines under examination [22] and, moreover, in both CpG and non-CpG dinucleotides (mCH, where H = A, C or T). Although the methylation patterns of CpG dinucleotides in ESCs and iPSCs were very similar, 1,175 DMRs were detected. The total length of individual DMRs was 1.68 Mb, varying from 1 to 11 kb per nucleotide. The distribution of DMRs over the genome was also heterogeneous: most DMR (80%) were associated with CG islands, 62% were near or inside the genes, and 29 and 19% were found within 2 kb from the transcription start or termination sites, respectively. Noteworthy, a group of shared DMRs was found in all the examined lines, in spite of the line-specific variations of the DMR number and localization. This fact attests to the existence of hot spots lacking epigenetic reprogramming, whose functions and roles in the genome remain poorly examined and need further analysis.

Moreover, the methylation patterns in the non-CpG regions were also different, although their general patterns in the genomes of ESCs and iPSCs were similar. A total of 29 non-CpG regions were detected [22]. The regions had a number of distinctive features: first, non- CpG-DMRs were rather extended: more than half of the DMRs were over 1 Mb long, and the total length of 29 DMRs was 32.4 Mb. Second, the genome localizations of non-CpG-DMRs and methylated CpG-DMRs were different: most non-CpG strongly biased towards centromeres and telomeres [22]. Notably, both K. Nishino *et al*. [20] and R. Lister *et al. *[21] detected 72 gene promoters undergoing differential methylation.

The DNA methylation profiles in five samples of mesenchymal stromal cells, eight different mesenchymal- derived iPSC lines, and three lines of human ESCs were compared using DNA microchips for a thorough analysis of the localization and dynamics of the CpG methylation in their genomes [23]. The genome-average methylation rate was 17 CpG sites per gene, with an average methylation percentage of CpG sites - 49.4, 70.6, and 70.5% in mesenchymal stromal cells, iPSCs from mesenchymal stromal cells, and ESCs, respectively. These data indicate that the reprogramming process tends towards the remodeling of semi-methylated regions into methylated ones. A total of 185,246 CpG sites were differentially methylated; 33,941 of them underwent further demethylation, while 151,306 became hypermethylated in the iPSCs. The CpG sites were further classified into groups, according to their localization in the genome: the CpG sites localized 1,500 or 200 bp upstream the transcription start point; in the 5’-non-translated regions; in the first exon; in the 3’-non-translated regions of the genes, and in the inter-gene regions [24]. The average methylation level increased during reprogramming in all regions; however, the methylation level of the promoters and first exon areas decreased; at that, the hypoand hypermethylated sites were located mainly in the inter-gene regions. In addition, the adjacent areas of CpG islands were analyzed as follows: 2 kb upstream or downstream a CpG island (shore regions), and 2 kblong regions flanking the shore regions (shelf regions). All other CpG sites were united into an open sea. In the mesenchymal stromal cells, the average methylation level of CpG islands was much lower (22.2%) than in the shore (67.5%) and shelf (42.7%) regions, and in the open seas (61.8%) [24]. These data indicate that reprogramming- associated changes in the DNA methylation pattern occurred mainly beyond the CpG islands. 3,744 ESC-iPSC DMRs were detected, 3,134 of them being hypermethylated and 610 being hypomethylated in the iPS cells as compared to ESCs [24]. It is interesting that the hypermethylated CpG sites in ESCs were localized mainly within 200 bp from the transcription start sites, in the first exons of the genes, and in the inter-gene regions, while in the iPSCs they localize 1,500 bp upstream the transcription start sites and in the intergene regions. A bioinformation analysis demonstrated that 610 hypermethylated CpG sites in iPSCs were associated with the genes involved in keratinization and keratin-differentiation processes, as well as epidermis cell differentiation and epidermis development.

Thus, the methylation profiles of iPSCs and ESCs are also different: in ESCs, highly methylated regions mainly localize in the proximal regions of gene promoters, while in the iPSCs - in the distal regions of gene promoters, inter-gene and open sea regions, as well as in the genes involved in epidermis development.

Interestingly, regular DMR distribution can be seen at the chromosome level as well: there are more X- chromosome-localized DMRs in the iPSCs carrying XX sex chromosomes than in the iPSCs with XY [20].

Therefore, the reprogramming of somatic cells into pluripotent ones is followed by the formation of DMRs, whose quantities vary depending on the initial cell type, reprogramming methods, culture conditions, etc. Most of these DMRs result from de novo aberrant methylation, while the smallest part is the consequence of epigenetic memory. Noteworthy, the formation of the DMRs resulting from the epigenetic memory is conditioned by both the initial type of somatic cells and the individual-specific patterns of DNA methylation in the cell donors. Special features of cell epigenomes were found even in monozygotic twins [23]. Part of these donor-specific epigenetic variations was unchanged during the reprogramming. For example, 1,129 differentially methylated CpG sites were detected using a comparative analysis of their methylation profiles in the iPSCs derived from the mesenchymal stromal cells of five different donors. These sites were associated mainly with the genes involved in the processing and presentation of antigens. The donor-specific DMRs localized mainly in gene bodies, the 3’-non-translated, and inter-gene regions [24].

Covalent histone modifications are involved in the maintenance of some epigenetic markers of initial-type somatic cells along with CpG methylation. Thus, in the iPSCs derived from β-cells of the human pancreas, the factor of PDX1 transcription was not repressed during re-programming. The method of chromatin immunoprecipitation demonstrated that an acetylated histone 3 associated with transcriptionally active chromatin is maintained in the promoters of the genes that encode insulin and PDX1 [15].

Thus, full-range genome-wide studies have demonstrated the presence of minimal differences in the patterns of DNA methylation, gene expression, and covalent histone modifications in these cells despite the close similarity among ESCs and iPSCs. One of the most topical issues is the impact of these differences on the properties of iPSCs.

## EFFECT OF THE EPIGENETIC MEMORY ON THE PROPERTIES OF INDUCED PLURIPOTENT STEM CELLS

The inherited features of the epigenomes and transcriptomes of the initial cell types affect only a small portion of genes. To what extent the aberrant regulation of these genes affects the properties of the resulting iPSCs is currently an issue of special interest. It has been established that DMRs inherited through epigenetic memory cause a shift in the differentiation spectrum; that is, the iPSC lines differentiate into somatic cells of the initial type. Thus, it was demonstrated that mouse iPSCs derived from either blood or skin cells possess different potentials of differentiation to either the hemopoietic or osteogenic direction, correspondingly. The iPSCs derived from blood cells more readily form hemopoietic colonies, while the iPSCs from skin cells form more colonies when differentiating in the osteogenic direction [16]. In addition, the differentiation potentials of human iPSCs from neonatal umbilical blood cells and foreskin keratinocytes have been assessed [21]. The expression levels of the early differentiation marker, the keratin-14 gene, were determined in embryoid bodies on the 6th day of culture. In iPSCs from keratinocytes, the expression of this gene was 9.4- fold higher, indicating a much higher differentiation potential for these cells towards keratinocytes as compared to that of iPSCs from the umbilical blood. This phenomenon is reciprocal: the differentiation potential of iPSCs from umbilical blood to hemopoiesis was much higher [21].

Another area where the epigenetical memory may cause serious problems is the use of iPSCs in *in vivo* studies. M. Stadtfeld *et al*. [25] examined murine iPSCs from various somatic progenitors: hemopoietic stem cells (11 lines), progenitor cells from the granulocytemacrophage line (11 lines), granulocytes (9 lines), peritoneal fibroblasts (6 lines), tail fibroblasts (6 lines), and keratinocytes (6 lines). The cells of the most newly established lines when in tetraploid complementation contributed poorly to chimaeras and failed to support the development of entirely iPSC-derived animals. A comparison of mRN As demonstrated that, in contrast to the ESC genes, the imprinted genes *Gtl2 *(or *Meg3*) and *Rian *of *Dlk1-Dio3 *locus proximal to the mouse* 12qF1 *were repressed both in most iPSC clones and in the initial somatic lines. It is common knowledge that the genes of this locus participate in the growth and differentiation of some tissues, as well as in postnatal neurological and metabolic processes [26]. A genomewide analysis of the microRN A expression profile demonstrated that the expression patterns of 21 of the 336 (6.3%) microRN As in ESCs differ from those in iPSCs, all of them being expressed from the *12qF1 *chromosome and repressed in iPSCs. The chromatin immunoprecipitation method has demonstrated that the acetylation levels of the H3 and H4 histones and that of methylated *H3K4 *associated with transcriptionally active chromatin are significantly lower in the iPSC* Dlk1-Dio3 *locus [25].

It is worth mentioning that not all the imprinted genes inherit the epigenetical and transcriptional statuses of initial somatic cell lines. Quantitative PCR demonstrated that the expression of the other imprinted genes is clone-specific [16]. This fact is supported by the results of another study with iPSCs from neutral stem cells isolated from a partenogenetic mouse embryo. In these cells, the expression levels of the cells with paternal imprinting, *Peg1 *(or *Mest*), *Ndn *and *Snurf *determined using microchips, was much lower than those in somatic cells from the embryos obtained by normal biparental fertilization, since these genes were reactivated during reprogramming [27]. Thus, the epigenetic memory phenomenon has a real impact on iPSC characteristics, and the consequences of its presence may be serious. Therefore, this aspect needs careful consideration when using iPSCs in disease modeling or in regenerative cell medicine.

## EFFECT OF CULTURE CONDITIONS AND CHEMICAL AGENTS ON THE EPIGENOME OF INDUCED PLURIPOTENT STEM CELLS

Minimal differences in the epigenomes and transcriptomes caused by the epigenetic memory or/and aberrant methylation de novo in PSCs and ESCs can result in rather significant changes in cells’ characteristics. Some logical questions emerge in this case: what are the factors affecting the type and number of these differences? Are there any artificial conditions that would allow one to correct these effects? Conditions and duration of culturing are the first noteworthy factors affecting iPSC quality in general and the number of epigenetic markers in particular. Reprogramming is a gradual process, and remodeling of the cell transcriptome and epigenome also takes a certain number of replication runs and mitoses, and, hence, the number of passages. The higher the number of passages, the lower the number of epigenetic differences is (if any). For example, in 12 independent lines of mouse iPSCs from various cell types (β-cells, fibroblasts, T-cells, and granulocytes), the number of differentially expressed genes varied at early passages from 500 to 2,000 depending on a line, and it decreased substantially to ~50 (and even to zero in some lines) after 14 passages [17]. The disappearance of differences among the iPSC lines correlated with the emergence of bivalent domains, trimethylated *H3K4 *(active chromatin marker) and* H3K27 *(inactive chromatin marker), typical of pluripotent cells [17]. A study of the methylation patterns in 7 independent lines of human iPSCs has also demonstrated a significant decrease in DMR in various lines from 80-256 at early passages to 30-70 at the 30th-40th passages [20]. A decrease in DMR numbers increases the ability of a line to differentiate into any of the three germ layers with equal effectiveness. For example, the effectiveness of the ability of iPSCs from keratinocytes to form hemopoietic colonies during differentiation was very low because of the residual methylation of the genes involved in hemopoiesis (e.g., *HOXD8*). The* HOXD8 *gene is significantly methylated in keratinocytes and, via the epigenetic memory, in iPSCs derived from them. The level of its methylation decreases during culturing, while the ability of cells to differentiate into hemopoietic cells simultaneously increases. However, this effect was observed only in one of two clones. Hence, long culturing of iPSCs might affect certain genome loci, but this was true not for the entire genome and not for all iPSC lines [21].

Two hypotheses can explain the elimination of the molecular and functional differences in iPSC clones during culturing. One of the possible mechanisms is the passive loss of the somatic markers associated with DNA replication. The alternative version is clone selection during culturing aimed at retention of the clones with fewer initial characters. However, a number of observations evidence against the selection. Thus, the proliferation levels and growth rates of clones from one cell are the same at early and late passages of iPSCs. The number of passages (that is, the required number of replication runs) necessary to eliminate inter-clone molecular and epigenetic differences also depends on the initial type of somatic cells [17].

Meanwhile, some findings attest to the lack of a decrease in the DMR number during culturing. For example, no changes in the DMR number at early (~15) and late (~65) passages were detected with a methylome analysis [22]. Culture conditions (medium composition, concentration of O_2_ and CO_2_, etc.) and/or the use of supplementary chemical agents are the other factors that could potentially affect the DMR number in iPSCs. The quality of iPSCs can be significantly improved by optimal conditions. Thus, the use of a medium supplemented with serum surrogate or with a mixture of embryonic bovine serum and serum surrogate instead of embryonic bovine serum alone provided an increase in the yield of clones in which the imprinted *Meg3 *gene from the *Dlk1-Dio3 *locus was reactivated [28].

Various chemical agents affect the gene expression as well. For example, treatment of mouse iPSCs with trichostatin (histone deacetylase inhibitor) and 5-azacytidine (DNA methylase inhibitor) causes changes in the epigenome [16]. Treatment of mouse iPSC clones in which the imprinted *Dlk1-Dio3 *locus was repressed with valproic acid (histone deacetylase inhibitor) caused reactivation of the locus genes. These iPS cells in tetraploid complementation could affect the development of an organism [25].

Ascorbic acid (vitamin C) also affects the DNA methylation pattern [29]. For example, dose-dependent reactivation of the imprinted *Meg3 *gene from the* Dlk1-Dio3 *locus was observed in iPSCs cultivated in an ascorbic acid-supplemented medium. However, ascorbic acid did not cause full-range demethylation of the entire genome; it could prevent aberrant demethylation of the *Dlk1-Dio3 *locus only, but it could not cause DNA demethylation in the stable clones of the iPSCs [28].

Meanwhile, the ability of cells to differentiate in a certain direction and their methylation profile can be restored by repeated reprogramming runs. For example, iPSCs from progenitors of a neural line had a very low ability to form colonies of hemopoietic cells. However, the reprogramming of these colonies significantly increased the formation of hemopoietic colonies by the secondary iPSCs [16]. Thus, iPSCs most closely similar to their standard, ESCs, can be obtained by varying the reprogramming system, culturing conditions, and duration, adding or removing chemical agents, etc. However, minimal differences in the transcriptomes and epigenomes of these cells still remain in any case. Is this factor a barrier for the practical use of iPSCs? This question is being currently discussed.

## USE OF THE EPIGENETIC MEMORY PHENOMENON IN BIOMEDICINE

Biomedicine, as well as pharmacology, needs new, more perfect, model systems of diseases. These models should meet certain criteria: repeatability, availability, usability, unambiguous result interpretation, adequate transferability (i.e., translation of the results of fundamental studies into practical medicine) [30-33].

The available array of studies in this area has demonstrated that the use of iPSCs is one of the most prospective approaches. However, in order to establish an iPSC-based model of a human disease, one should consider all factors that could potentially affect the quality of the results. Epigenetic memory is one of the significant factors. Is this phenomenon an advantage or a disadvantage of iPSC-based models of human diseases? This is a pending issue. Let us consider the problems of modern medicine in the context of using iPSCs and try to solve one of these problems.

The availability of certain cell material suitable for study is the first urgent problem in cell replacement therapy. This problem can be subdivided further. First of all, it is associated with the availability of initial donor cells: this may be a problem, since obtaining biopsy material for many types of cells (e.g., neurons or epithelium of the internals) is a challenge. The second problem is the quantity of the available material, which is limited even when biopsy is available. Moreover, the cells are usually terminally differentiated, and, hence, their proliferative activity is limited. Therefore, all full-scale manipulation analyses cannot be performed using conventional methods. iPSCs obtained from a limited biopsy mass can solve the problem. Their proliferative potential is unlimited; therefore, they can be repeatedly differentiated into cells of the required type, providing thus an unlimited cell source for all relevant analyses and manipulations.

**Figure:**
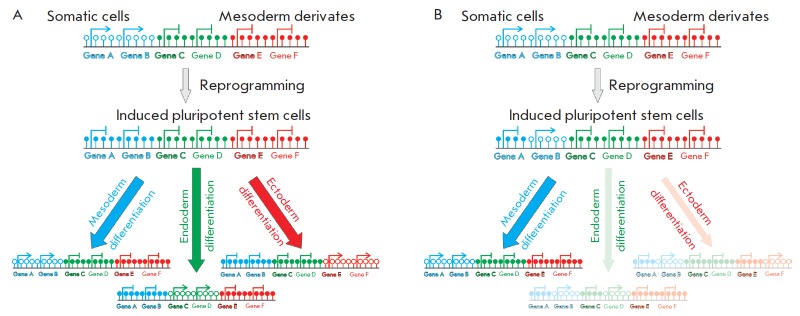
“Epigenetic memory” phenomenon in induced pluripotent stem cells. A – The “ideal” reprogramming process of somatic cells to pluripotency: differentiation of induced pluripotent stem cells into each of the germ layers is an equally likely event. B – As a result of the re-programming process induced pluripotent stem cells can retain some features of the epigenome of the donor tissue. This phenomenon shifts the differentiation: induced pluripotent stem cells preferentially generate derivates of the donor somatic cell type

The next problem is correct and efficient differentiation of iPSCs into cells of a desired type. The protocols of targeted differentiation are available now for a limited number of cell cultures, although current information on signaling pathways and transcription factors related to development into a certain direction is plentiful. Therefore, even the availability of iPSC lines does not guarantee the obtaining of a certain narrowly specialized cell type. This problem could be solved by the phenomenon of epigenetic memory. We suggest the following scheme for using this phenomenon for cell replacement therapy (see *Figure*). It is well known that epigenomes and transcriptomes of the initial cell type maintained in iPSCs make them differentiate into somatic cells of the initial type. Hence, it would be reasonable to use the biopsy material of cells of the same origin. A number of issues should be considered in this case: first, ten or more iPSC clones should be analyzed to choose the most optimal clones from the variances. Second, the overall transcriptome and methylome data must be compared with the available databases; this will allow a scientist to detect the so-called hot spots of underreprogramming that emerge via gene reactivation during the re-programming, and the spectra of the genes with epigenetic markers inherited from the somatic cells of the progenitor type. Finally, the direction of cell differentiation could be predicted or changed, by special means, to a desirable one, after the genes affected by the epigenetic memory are examined at the functional level. Thus, this case allows us to demonstrate that the disadvantages of iPSC, such as the inheritance of a number of epigenome and transcriptome features caused by invalid reprogramming, can be converted into advantages.

## Abbreviations

ESCs: embryonic stem cells
iPSCs: induced pluripotent stem cells
DMR: differentially methylated regions
